# The cardiovascular safety of tricyclic antidepressants in overdose and in clinical use

**DOI:** 10.1177/20451253241243297

**Published:** 2024-05-30

**Authors:** David Taylor, Sofia Poulou, Ivana Clark

**Affiliations:** Pharmacy Department, Maudsley Hospital, South London and Maudsley NHS Foundation Trust, Denmark Hill, London SE5 8AZ, UK; Institute of Pharmaceutical Sciences, King’s College London, London, UK; Institute of Pharmaceutical Sciences, King’s College London, London, UK; Institute of Pharmaceutical Sciences, King’s College London, London, UK; Pharmacy Department, Maudsley Hospital, London, UK

**Keywords:** cardiac events, overdose, TCA, toxicity, tricyclic antidepressant

## Abstract

Tricyclic antidepressants (TCAs) remain widely prescribed for depression and many other conditions. There may be important differences between individual TCA in regard to their overdose toxicity and their cardiac toxicity in clinical use. We conducted a systematic review to compare the toxicity of individual TCA in overdose and the risk of serious adverse cardiac events occurring with therapeutic doses. We used the fatal toxicity index (FTI) and case fatality ratio as markers of fatality in overdose, and hazard ratios or odds ratios for the risk of cardiovascular adverse events during normal clinical use. In all, 30 reports of mortality in overdose and 14 observational studies assessing the risk of cardiovascular adverse events in clinical use were included. FTI values were of the same order of magnitude (10^1^–10^2^) for all TCAs except lofepramine. Desipramine appears to be somewhat more likely than other TCAs to lead to death in overdose. Amitriptyline, clomipramine, dothiepin/dosulepin, doxepin, trimipramine and imipramine showed broadly similar toxicity and were usually reported to be less toxic than desipramine. Data on nortriptyline were contradictory. Lofepramine had the lowest risk of death in overdose. The rank order of overdose toxicity was broadly consistent between different FTI definitions and between markers used. With respect to the risk of cardiovascular events at clinically relevant exposure, amitriptyline, nortriptyline and lofepramine were associated with a greater risk of in-use cardiotoxicity. All measures of overdose toxicity were subject to external influences and confounding. The continued use of TCAs in depression and other conditions should be minimized when considering their undoubted toxicity in overdose and possible toxicity in normal clinical use.

## Plain language summary

Older tricyclic antidepressants and their toxicity in overdose and in clinical use

Tricyclic antidepressants were first used in the 1950s. Their use for depression has gone down in the past 20 or so years. This is because newer antidepressants are better tolerated and less toxic in overdose. Certain tricyclics - dosulepin and amitriptyline - have been identified as being particularly toxic tricyclics and their use has been restricted. However many other tricyclics remain widely used for depression and many other conditions. We examined all the evidence we could find on tricyclic toxicity. We found that, with one exception, all tricyclics are toxic and dangerous when taken in overdose. The exception is lofepramine - a tricyclic used in the UK and some other countries. When looking at toxicity in clinical use, we found no consistent evidence of difference between individual tricyclics. It is possible that most or all tricyclics do not increase the risk of heart attack or sudden cardiac death when used at normal clinical doses.

## Introduction

Depression is a common condition strongly associated with suicide, cardiovascular disease (CVD) and increased cardiovascular mortality.^1^ Antidepressants given to treat depression are frequently used in suicide attempts either alone or in combination with other drugs or alcohol and have been also associated with a range of adverse cardiovascular effects.^1^ These adverse cardiovascular effects are considered responsible for some of the mortality associated with antidepressant overdose in suicide attempts,^2^ although seizures, respiratory depression and coma also contribute to the overall risk of death.^3–5^ Among TCAs, there appear to be important differences in the relative toxicity of individual drugs.^1^ Perhaps the best-known example is the striking difference in overdose toxicity between desipramine and a prodrug of desipramine, lofepramine.

TCAs are associated with a range of adverse cardiovascular effects even at therapeutic doses. These include an increase in heart rate, orthostatic hypotension and cardiac conduction abnormalities. Adverse cardiovascular effects associated with TCAs have been reported in patients with CVD but also in people without previous CVD.^6–12^ There have also been a number of case reports of sudden unexplained death reported in children receiving TCAs.^13–16^ These cardiac effects are more pronounced in overdose and, partly as a result, TCAs are the class of antidepressants most commonly associated with death from suicides.^17,18^

The mechanism through which TCAs cause adverse cardiovascular effects is complex. They have significant activity as antagonists of α_1_-, H_1_-, H_2_- and M-receptors and block Na^+^ and human ether-a-go-go-related gene channels. The most common arrhythmia induced by TCAs is sinus tachycardia. This is because TCAs have anticholinergic activity and cause inhibition of norepinephrine uptake. Bradyarrhythmias (due to atrioventricular block) and tachyarrhythmias (supraventricular and ventricular) may also occur.^2,19^ Orthostatic hypotension is a very common adverse effect of TCAs mainly caused by a combination of reduced myocardial contractility and reduced systemic vascular resistance owing to alpha-adrenergic blockade.^2,20–23^ TCAs have been also shown to exert detrimental effects on cardiac sympathetic control, leading to hypertension.^2,21,24–26^ An increased risk of myocardial infarction (MI) has been also reported in association with TCAs, consistent with their effects on cardiac conduction and heart rate.^27^

TCA overdose may result in death by various mechanisms. Life-threatening complications resulting from delayed cardiac conduction include complete heart block and ventricular re-entry arrhythmias.^2,11,21,22,28–30^ TCAs also reduce seizure threshold and this mechanism may also contribute to overdose mortality,^31^ along with respiratory depression and hypotension.^3^

In general, selective serotonin reuptake inhibitors are now preferred to TCAs because they are associated with less severe adverse effects and lower mortality in overdose, and clinical practice guidelines vary in their recommendations on the use of TCAs. Nonetheless, TCAs are still widely prescribed for depression and are still considered important drugs in the armamentarium of antidepressants.^32–37^ Moreover, TCAs are today very widely used in medicine^38^ for conditions such as neuropathic pain,^39^ migraine,^40^ chronic tension-type headache,^41^ insomnia,^42^ smoking cessation^43^ and nocturnal enuresis.^44^ Thus, knowledge of the relative cardiovascular safety of individual TCAs remains highly clinically relevant.

In the past 10 or 20 years, some national authorities have sought to minimize the prescribing of what were considered to be the two most toxic TCAs, amitriptyline and dothiepin/dosulepin,^45,46^ while issuing no warnings against the use of other TCAs. In this analysis, we sought to discover the difference in toxicity of a range of TCAs, to examine whether or not this distinction is valid and to establish the relative safety of individual drugs in clinical use and overdose.

## Methods

We performed a comprehensive review of the literature to compare the relative toxicity of different TCAs both in terms of the risk of mortality associated with overdose and the risk of adverse events associated with therapeutic doses, using data from large epidemiological studies.

We followed the Preferred Reporting Items for Systematic Reviews and Meta-Analysis checklist for systematic reviews.^47^

We searched the literature to identify retrospective and prospective observational studies assessing:

(a) Mortality data for each TCA including measures of either fatal toxicity index (FTI) and case fatality rate (CFR).^48^
(b) The risk [hazard ratio (HR), odds ratio (OR) or absolute risk (AR)] of heart failure, MI, stroke/transient ischaemic attack (TIA), arrhythmia or other cardiac death associated with the therapeutic use of each TCA included.

The TCAs included in the review were as follows:

• amitriptyline
• clomipramine
• desipramine
• dothiepin (dosulepin)
• doxepin
• imipramine
• lofepramine
• nortriptyline
• trimipramine

We defined TCAs chemically as dibenzazepines, dibenzocycloheptadienes, dibenzoxepins and dibenzothiepines. We included what we considered to be widely used TCAs, broadly defined as TCAs at some time licensed in the UK (from where most TCA toxicity and safety data are reported) for more than a decade. Excluded TCAs included butriptyline, dibenzepin, loxapine, opipramol and protriptyline – TCAs rarely or never used in the UK.

Studies were included in the review if they directly calculated any of the markers listed above or if they provided data from which the markers could be calculated. When available, we checked FTI calculations from raw data. We searched for indications of data certainty (e.g. confidence intervals), statistical comparisons reported in individual studies and recorded outcomes for reporting in the systematic review. We did not perform any original statistical comparisons. We also examined overlap in time and geography for the studies identified to identify data that might have contributed to more than one study.

We searched PubMed in December 2020 without language or date restrictions using the following key words:

((Antidepressive Agents OR Antidepressants OR Antidepressant OR Tricyclic OR amitriptyline/Saroten/Tryptanol/Tryptizol OR desipramine/Pertofran OR clomipramine/Anafranil OR doxepin/Sinequan OR dothiepin/dosulepin/Prothiaden OR imipramine/Tofranil OR lofepramine/Gamonil/Gamanil OR nortriptyline/Nortrilen/Allegron/Noritren OR trimipramine/Surmontil)) AND (heart failure OR myocardial infarction OR stroke OR transient ischaemic attack OR transient ischemic attack OR arrhythmia OR death OR fatal OR fatality). All retrieved papers were scrutinized for other relevant publications.

The initial search and selection were conducted by one of us (SP) and the selection process was independently repeated by another (IC) after the identification of full papers for review. A third author (DT) was available to resolve differences in selection that could not be agreed upon by the two independent searchers. The search was repeated in November 2023 exactly as before using PubMed, but also including the search of EMBASE (*via* OVID). Search terms were adapted to the thesaurus of each database (e.g. for EMBASE: exp antidepressant agent/ or exp human AND fatal toxicity index.mp. OR exp case fatality rate/).

Potentially relevant publications were identified through the electronic search conducted in December 2020. These publications were screened to identify observational studies that assessed mortality data for individual TCAs in overdose or the risk of cardiovascular adverse events associated with individual TCAs in clinical use.

## Results

**Figure 1.**
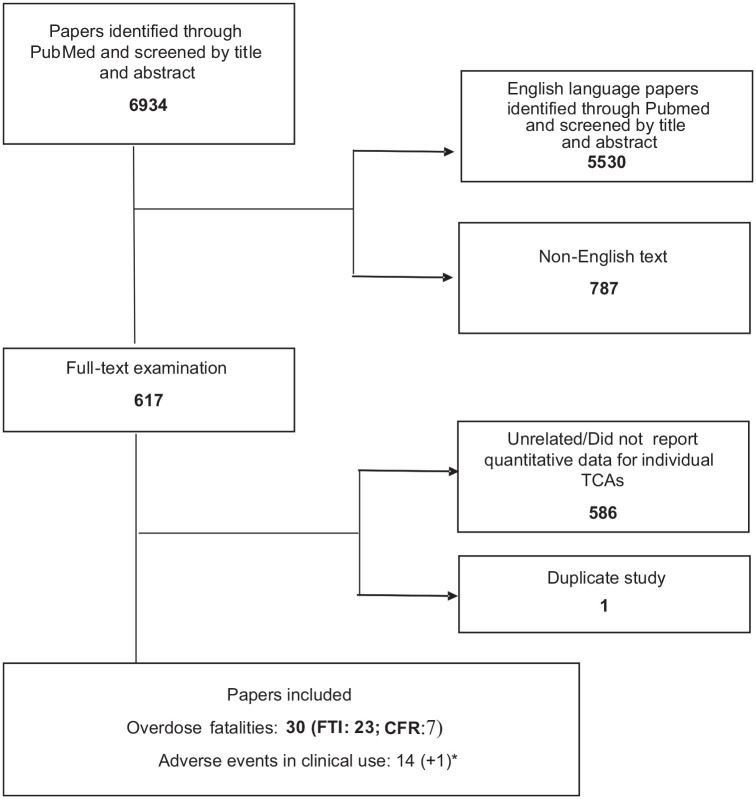
Flow chart of the selection of studies for inclusion in the review (*found in the second search). CFR, case fatality rate; FTI, fatal toxicity index; TCAs, tricyclic antidepressants.

The original search outcome is illustrated by the flow diagram (Figure 1). After applying the search strategy according to the protocol, 6934 studies were identified for screening; 5530 did not fit the inclusion criteria (either not in English or not including interpretable data on mortality or clinical toxicity relating to individual TCAs). Of these, 617 studies were selected for full-text examination. After full-text examination, we identified 30 trials assessing mortality data in overdose (FTI: 23; CFR: 7) and 14 observational studies assessing the risk of adverse cardiovascular events in clinical use, which were included in the review. There were no differences in selection so no third-party resolution was required. One additional paper^49^ was uncovered in our November 2023 search.

### Fatal toxicity index

Four different definitions for FTI were identified: deaths per million prescriptions; deaths per million patients; deaths per consumption [per million defined daily doses (DDD) or DDD per 1000 inhabitants (inh)/year] and deaths per 10,000 treatment episodes. Of the 23 studies, 14 assessed FTI as the number of deaths per million prescriptions,^17,48,50–61^ 2 studies as the number of deaths per million patients,^62,63^ 8 studies as the number of deaths per consumption^55,61,64–69^ and 1 study as the number of deaths per 10,000 treatment episodes^70^ (Table 1). Two of these studies included both deaths per million prescriptions and deaths per consumption (Table 1).

**Table 1.** Characteristics of studies assessing FTI.

Authors	Definition of FTI	Drug exposure	Region	Years
Buckley and McManus, 1998^50^	Deaths per million prescriptions	Single	England and Wales	1983–1992
Buckley and McManus, 2002^51^	Deaths per million prescriptions	Single	Scotland, England and Wales	1993–1999
Cassidy and Henry, 1987^52^	Deaths per million prescriptions	Single	Scotland, England and Wales	1975–1984
Cheeta *et al*., 2004^53^	Deaths per million prescriptions	Both single and multiple	England and Wales	1998–2000
Farmer and Pinder, 1989^54^	Deaths per million prescriptions	Single and both single and multiple	England and Wales	1979–1985
Frey *et al*., 2000^64^	Deaths per consumption (deaths/10^6^ DDD)	Single and multiple	Austria	1991–1997
Fountain *et al*., 2020^55^	Deaths per million prescriptions and deaths per consumption (deaths/10^6^ DDD)	Single and multiple but ‘primary contributor’ identified	New Zealand	2008–2013
Hawton *et al*., 2010^48^	Deaths per million prescriptions	Single	England and Wales	2000–2006
Henry, 1989^56^	Deaths per million prescriptions	Single	Scotland, England and Wales	1982–1986
Henry and Antao, 1992^57^	Deaths per million prescriptions	Single	Scotland, England and Wales	1985–1989
Henry *et al*., 1995^58^	Deaths per million prescriptions	Single	Scotland, England and Wales	1987–1992
King and Moffat, 1983^59^	Deaths per million prescriptions	Both single and multiple	England and Wales	1976–1978
Leonard, 1986^62^	Deaths per million patients	Single	England and Wales	1977–1983
Malmvik *et al*., 1994^69^	Deaths per consumption (DDD/1000 inh/year)	NR	Sweden	1986–1989
Mason *et al*., 2000^70^	Deaths per 10,000 treatment episodes	Single and both single and multiple	England and Wales	1993–1995
Montgomery and Pinder, 1987^63^	Deaths per million patients	Single	England and Wales	1977–1984
Morgan *et al*., 2004^60^	Deaths per million prescriptions	Single and both single and multiple	England	1993–2002
Ohberg *et al*., 1998^65^	Deaths per consumption (DDD/1000 inh/year)	Primary agent^a^	Finland	1990–2000
Ojanperä *et al*., 2016^66^	Deaths per consumption (deaths/10^6^ DDD)	Primary agent^a^	Finland	2005, 2009, 2013
Reis *et al*., 2007^67^	Deaths per consumption (deaths/10^6^ DDD)	Single	Sweden	1992–2005
Reith *et al*., 2003^61^	Deaths per million prescriptions and deaths per consumption (deaths/10^6^ DDD)	Primary agent^a^ and both single and multiple	New Zealand	2001
Shah *et al.*, 2001^17^	Deaths per million prescriptions	Both single and multiple	England	1993–1997
Vuori *et al*., 1989^68^	Deaths per consumption (DDD/1000 inh/year)	Primary agent^a^	Finland	1985–1987

a Primary agent = drug with the greatest concentration in blood in relation to its expected therapeutic concentration.

DDD, defined daily doses; FTI, fatal toxicity index; NR, not reported.

Some studies included deaths only from single-drug exposures (i.e. where only one causative drug was identified pre or postmortem), whereas others included deaths from both single- and multiple-drug exposures. We analysed these separately.

The definition of FTI, the type of drug exposure (i.e. single exposure only or both single exposure and multiple exposure) and the region and years covered by each study are presented in Table 1.

The FTIs reported for each TCA in the studies included in this review as deaths per million prescriptions from single-drug exposures and from single- and multiple-drug exposures are presented in Tables 2 and 3.

**Table 2.** Estimated FTI: ^a^Deaths per million prescriptions from single-drug exposures.

	Buckley and McManus, 1998^50^	Buckley and McManus, 2002^51^	Cassidy and Henry, 1987^52^	Farmer and Pinder, 1989^54^	Fountain *et al*., 2020^55^	Hawton *et al.*, 2010^48^ ^b^	Henry and Antao, 1992^57^	Henry, 1989^56^	Henry *et al*., 1995^58^	Morgan *et al*., 2004^60^	Reith *et al*., 2003^71^ ^c^
Amitriptyline	61 (57.2–64.7)	38 (35.5–40.5)	47 (43.9–49.1)	64	17 (11.4–25.3)	11 (10.3–12.6)	50	56	39 (35.63–42.39)	28 (26.2–30)	21 (6.7–48.5)
Clomipramine	11 (8.3–14.6)	13 (9.4–16.3)	11 (8.1–14.1)	15	93 (38.5–222.3)	14 (10–19.3)	9	10	7 (4.74–10.32)	13 (10.2–16.8)	36 (0.9–201)
Desipramine	123 (49.3–252.6)	201 (92–381.6)	80 (36.6–123.8)				63	149	76 (14.28–185.74)		0 (0–139.10)
Dothiepin/dosulepin	68 (64.2–71.5)	53 (50.5–56.1)	50 (45.8–54.2)	82	62 (36.5–104.0)	36 (33.4–39.3)	53	60	48 (44.60–51.23)	49 (46.1–51)	66 (26.4–135.5)
Doxepin	50 (40.7–61.7)	25 (18–34.3)	31 (25.2–37.4	57	39 (19.3–77.3)	28 (17.6–42.6)	34	41	24 (16.89–32.35)	21 (14.9–29.7)	20 (2.4–71.4)
Imipramine	41 (35.1–46.4)	33 (27–39.5)	28 (25.1–31.7)	43		12 (8.1–18.4)	33	30	32 (25.95–37.68)	28 (23.3–32.1)	36 (0.9–199.6)
Lofepramine	3 (1.5–5.9)	1 (0.6–2.4)							2 (1.15–4.14)		
Nortriptyline	60 (42.5–83.1)	6 (2.2–11.4)	39 (29.0–39.4)		28 (17.9–44.0)	10 (3.2–23.2)	54	42	52 (31.11–77.67)	1^d^ (0.3–1.2)	60 (19.6–140.7)
Trimipramine	26 (20.6–31.4)	17 (11.7–22.5)	28 (23.3–31.9)	51		15 (8–25.6)	25	30	14 (9.64–19.01)	13 (CI: 10–18.2)	30 (0.8–168.8)

a Rounded to the nearest integer.

b Not necessarily single-drug exposure; primary agent involved in the fatality.

c Deaths only from suicide or undetermined intent.

d Possibly a miscalculation – the figure is 6 (5.47) if calculated from data given in the report.

CI, confidence interval; FTI, fatal toxicity index.

**Table:** Estimated FTI: ^a^deaths per million prescriptions from single-drug exposures – study overlap analysis.

UK	
Study	Time period
Cassidy and Henry, 1987^52^	1975–1984
Henry and Antao, 1992^57^	1975–1989
Henry, 1989^56^	1982–1986 (updated since Cassidy and Henry, 1987)
Farmer and Pinder, 1989^54^	1979–1985
Henry *et al.*, 1995^58^	1987–1992
Buckley and McManus, 1998^50^	1983–1992
Buckley and McManus, 2002^51^	1993–1999
Morgan *et al.*, 2004^60^	1993–2002
Hawton *et al.*, 2010^48^	2000–2006

Shading = overlap of data collection periods.

**Table:** 

New Zealand	
Study	Time period
Reith *et al.*, 2003^61^	2001
Fountain *et al.*, 2020^55^	2008–2013

**Table 3.** Estimated FTI: ^a^Deaths per million prescriptions from studies not distinguishing between single-drug and multiple-drug exposures.

	Cheeta *et al.*, 2004^53^ [SPMR (95% CI)]**	Farmer and Pinder, 1989^54^	King and Moffat, 1983^59^	Morgan *et al.*, 2004^60^ (95% CI)	Reith *et al.*, 2003^61^	Shah *et al.*, 2001^17^ (95% CI)
Amitriptyline	15 [1.8 (1.5–2.1)]****	104	80	43 (41.0–45.4)	42 (19.9–76.4)	61 (57–66)
Clomipramine	11 [1.3 (0.7–2.1)]	48	43	24 (20.6–28.4)	36 (0.9–201)	23 (18–30)
Desipramine	0 (but only 300 prescriptions)				377 (9.6–2101.7)	550 (345–833)
Dothiepin/dosulepin	14 [1.7 (1.5–2.0)]****	116	77	65 (62.2–67.9)	113 (58.7–197)	76 (72–80)
Doxepin	3 [0.3 (0.0–1.2)]	95	79	34 (25.3–43.7)	20 (2.4–71.4)	38 (27–54)
Imipramine	11 [1.4 (0.7–2.4)]	83	64	42 (36.2–49.4)	36 (0.9–199.6)	54 (45–65)
Lofepramine	1 [0.1 (0.0–0.4)]****					7 (5–1)
Nortriptyline	9^b^ [1 (0.2–2.5)]		35	10^c^ (5.4–16.0)	60 (19.6–140.7)	63 (47–83)
Trimipramine	6 [0.7 (0.2–1.6)]	97	71	26 (20.6–33.2)	61 (7.3–218.8)	33 (25–43)

a Rounded to the nearest integer.

b Excludes 10 cases where both amitriptyline and nortriptyline were mentioned together.

c Excludes 70 cases where both amitriptyline and nortriptyline were mentioned together.

** Standardized proportionate mortality ratio (SPMR) (95% CI).

**** *p* < 0.0001 for total expected deaths in comparison with total observed deaths (as reported by the original study).

CI, confidence interval; FTI, fatal toxicity index.

**Table:** Estimated FTI: deaths per million prescriptions from single and multiple-drug exposures – overlap analysis.

Paper	Time period
King and Moffat, 1983^59^	1976–1978
Farmer and Pinder, 1989^54^	1979–1985
Shah *et al.*, 2001^17^	1993–1997
Morgan *et al.*, 2004^60^	1993–2002
Cheeta *et al.*, 2004^53^	1998–2000

Shading = overlap of data collection periods.

**Table:** 

New Zealand	
Reith *et al.*, 2003^61^	2001

FTIs as calculated by other definitions identified are presented in Tables 4–6.

**Table 4.** Estimated FTI: ^a^Deaths per consumption of primary agent considered responsible for death.

	Frey *et al*., 2000^64^ ^b^	Fountain *et al*., 2020^55^ ^b^ (95% CI)	Malmvik *et al*., 1994^69^ ^c^	Ohberg *et al*., 1998^65^ ^c^ (95% CI)	Ojanperä *et al*., 2016^66^ ^b^	Reis *et al*., 2007^67^ ^b^	Reith *et al*., 2003^61^ ^b^ (95% CI)	Vuori *et al*., 1989^68^ ^c^
Amitriptyline	12.1***	0.6 (0.4–0.9)	24.0	12.1 (10.9–13.2)	6.5	1.8	3.0 (1.43–5.47)	4.0
Clomipramine	3.6	1.7 (0.7–4.0)	7.5	8.3 (5.7–11.4	4.3	0.4	2.0 (0.05–10.10)	
Desipramine							16.9 (0.43–94)	
Dothiepin/dosulepin	1.2*	1.7 (1.0–2.9)					8.5 (4.41–14.90)	
Doxepin	18.6***	1.1 (0.6–2.2)		21.3 (19.5–23.6)	14		1.4 (0.17–5.12)	6.4
Imipramine			12.5			0.6	2.2 (0.06–12.5)	
Lofepramine			0.7					
Nortriptyline	15.0	0.9 (0.6–1.4)	6.7		1.7	1.3	3.3 (1.08–7.74)	
Trimipramine			32.4	17.2 (11.8–24.2)	6.3	1.9	5.1 (0.62–18.3)	

a Rounded to the first decimal place.

b Deaths/10^6^ DDD.

c Deaths/DDD/1000 inh/day.

*** *p* < 0.001 (significantly different from all antidepressants by *x* test). *p < 0.05 (as reported by the original study).

CI, confidence interval; FTI, fatal toxicity index.

**Table:** Estimated FTI: deaths per consumption of primary agent considered responsible for death from different studies – study overlap analysis.

Paper	Time period	Country
Frey *et al*., 2000^64^	1991–1997	Austria
Fountain *et al.*, 2020^55^	2000–2013	New Zealand
Reith *et al*., 2003^61^	2001	New Zealand
Malmvik *et al.*, 1994^69^	1986–1989	Sweden
Reis *et al.*, 2007^67^	1992–2005	Sweden
Ohberg *et al.*, 1998^65^	1990–1995	Finland
Ojanperä *et al*., 2016^66^	2005, 2009 and 2013	Finland
Vuori *et al*., 1989^68^	1985–1987	Finland

Shading = overlap of data collection periods.

The studies assessing FTI as deaths per million patients and deaths per 10,000 treatment episodes are described in Tables 5 and 6.

**Table 5.** Estimated FTI: deaths per million patients.

	Leonard, 1986^62^	Montgomery and Pinder, 1987^63^
Amitriptyline	166	166
Clomipramine	34	32
Dothiepin/dosulepin	147	143
Doxepin	99	106
Imipramine	105	106
Trimipramine	93	87

FTI, fatal toxicity index.

**Table:** Study overlap analysis.

Paper	Time period	Country
Leonard, 1986^62^	1977–1983	UK
Montgomery and Pinder, 1987^63^	1977–1984	UK

Shading = overlap of data collection periods.

**Table 6.** Estimated FTI: ^a^Deaths per 10,000 treatment episodes from Mason *et al*., 2000.^70^

	Mason *et al.*, 2000^70^	
	Single-drug exposure	Single- and multiple-drug exposure
Amitriptyline	1.6	2.2
Clomipramine	0.4	0.9
Desipramine	6.1	18.4
Dothiepin/dosulepin	1.4	1.8
Doxepin	1.1	2.0
Imipramine	1.8	2.5
Lofepramine	0.0	0.2
Nortriptyline	0.8	3.8
Trimipramine	1.4	2.1

a Rounded to the first decimal place.

FTI, fatal toxicity index.

### Case fatality rate

The characteristics of the studies assessing CFR are presented in Table 7.

**Table 7.** Characteristics of studies assessing CFR.

	Method of calculation	Drug exposures (single, multiple or both)	Region	Years
Alsén *et al*., 1994^72^	Completed suicides/suicide attempts	Both single- and multiple-drug exposures	Lund and Orup, Sweden	1987–1990
Amitai and Frischer, 2004^73^	Number of deaths/number of exposures (%)	Primary agent^a^	USA	1983–2002
Hawton *et al.*, 2010^48^	Mortality rate/self-poisoning rate (%)	Single	England and Wales	2000–2006
Nelson and Spyker, 2017^74^	Number of deaths/number of exposures (%)	Single	USA, Puerto Rico and the District of Columbia	2000–2014
Pfeifer *et al*., 2020^75^	Completed suicides/attempted suicides	‘Leading suicide method’	Switzerland	2000–2010
Rose and Unis, 2000 (average values)^26^	Number of deaths/number of overdoses (%)	Primary agent^a^	USA	1989–1997
White *et al.*, 2008^76^	Mortality/suicidal ingestions (%)	Single	USA	2000–2004

a Primary agent = drug assigned as the causative agent.

CFR, case fatality rate; NR, not reported.

The results of studies assessing CFRs for individual TCAs are presented in the following Table 8.

**Table 8.** Estimated case fatality index (CFR)^a^: number of deaths/number of exposures (%).

	Amitai and Frischer, 2004^73^ ^b^	Alsén *et al*., 1994^72^ ^b^	Hawton *et al*., 2010^48^ ^b,c^	Nelson and Spyker, 2017^74^ ^c,d^ (95% CI)	Pfeifer *et al*., 2020^75^ (95% CI)	Rose and Unis, 2000^26^ (95% CI)	White *et al.*, 2008^76^ ^b,c^
Amitriptyline	0.7	10.4	0.9	0.4 (0.31–0.44)	0.04 (0.02–0.09)	0.9 (0.81–0.2)	0.7
Clomipramine		2.3	1.3	0.0 (0–0.2)	0.2 (0.05–0.4)		0.5
Desipramine	1.6			1.4 (0.7–2.5)		1.9 (1.3–2.4)	3.2
Dothiepin/dosulepin			2.3				
Doxepin			2.3	0.5 (0.4–0.73)	0.4 (0.2–0.6)	0.8 (0.5–1)	1.1
Imipramine	0.6	0.0	1.3	0.4 (0.21–0.71)		0.7 (0.5–1)	1.9
Lofepramine		0.0					
Nortriptyline	0.7		1.1	0.3 (0.2–0.5)		0.9 (0.7–1)	0.3
Trimipramine		18.4	1.4	0.0 (0–7.3)	0.2 (0.1–0.2)		0.0

a Rounded to one decimal place.

b Calculated from population data provided in the study report.

c Single-drug exposures.

d Exposures may involve suspected suicide attempts but also may include other reasons such as unexpected adverse events, therapeutic errors or other forms of intentional or unintentional misuse.

**Table:** Estimated case fatality index (CFR)^a^ – study overlap analysis.

Paper	Time period	Country
Amitai and Frischer, 2004^73^	1983–2002	USA
Alsén *et al*., 1994^72^	1987–1990	Sweden
Hawton *et al*., 2010^48^	2000–2006	UK
Nelson and Spyker, 2017^74^	2000–2014	USA, Puerto Rico and the District of Columbia
Pfeifer *et al*., 2020^75^	2000–2010	Switzerland
Rose and Unis, 2000^26^	1989–1997	USA
White *et al.*, 2008^76^	2000–2004	USA

a Rounded to one decimal place.

### Toxicity during clinical use

Table 9 depicts the characteristics of the studies identified that reported risk data for the selected adverse events. These include the study design, the population included, the adverse cardiovascular events and the region where the study was conducted.

**Table 9.** Characteristics of studies assessing adverse cardiovascular events in clinical use.

	Design	Population	Events of interest studied	Region
Alqdwah-Fattouh *et al*., 2020^77^	Nested case–control	Aged 40–99	MI	Spain
Biffi *et al*., 2018^19^	Nested case–control and case-crossover	Elderly affected by a previous CVD	Arrhythmia (risk of hospitalization for arrhythmia in the elderly)	Italy
Brouwers *et al.*, 2016^78^	NR	Patients with heart failure	All-cause mortality Cardiovascular mortality	Denmark
Coupland *et al*., 2011^79^	Cohort study and nested self-controlled case-series	Elderly (⩾65 years)	Stroke/TIA MI All-cause mortality	UK
Coupland *et al.*, 2016^80^	Self-controlled case series	People aged 20–64	Arrhythmia MI Stroke/TIA	UK
Danielsson *et al*., 2016^81^	Matched case control	Elderly (⩾65 years)	All-cause mortality	Sweden
Eroglu *et al*., 2022^49^	Nested-case control	General population	OHCA	Denmark
Jolly *et al*., 2009^82^	Matched case control	General population	Sudden death – selection of cases aimed at identifying arrhythmic deaths	Midlands of England
Leonard *et al*., 2011^83^	Cohort study	General population	Sudden cardiac death or ventricular arrhythmia relative to paroxetine	California, Florida, New Ohio and Pennsylvania
Spindelegger *et al*., 2014^84^	Cohort study	General population	Arrhythmia (only severe ADRs)	Institutions in Switzerland, Germany and Austria
Tata *et al.*, 2005^85^	Case control	General population	First MI	Great Britain and Ireland
Wang *et al.*, 2015^86^	Nested case control	Patients who survived a first hospitalization with stroke	Stroke recurrence	Taiwan
Weeke *et al.*, 2012^87^	Case-time-control	General population	OHCA	Denmark
Wu *et al*., 2017^88^	Case-crossover	General population	Hospitalization for AMI	Taiwan
Wu *et al.*, 2017^88^	Cohort study	Patients with depressive disorders	Sudden cardiac death or ventricular arrhythmia relative to paroxetine	Taiwan

ADRs, adverse drug reactions; CVD, cardiovascular disease; MI, myocardial infarction; OHCA, out-of-hospital cardiac arrest; TIA, transient ischaemic attack.

The results of studies assessing the relative risk of stroke/TIA are presented in Supplemental Table S1. Of the TCAs studied only lofepramine [HR 1.26 (CI: 1.02–1.54)]^79^ and imipramine [HR 1.41 (CI: 1.13–1.76)]^86^ were associated with increased risk of stroke in clinical use.

Supplemental Table S2 presents associations between TCA use and arrhythmia. Of the TCAs studied only lofepramine [2.13 (CI: 1.05–4.33)],^80^ [1.67 (CI: 1.01–2.76)]^80^ was associated with increased risk of arrhythmia.

Two studies assessed the risk of out-of-hospital cardiac arrest (OHCA) with individual TCAs. One looked at amitriptyline, nortriptyline and imipramine and found only nortriptyline [5.14 (CI: 2.17−12.2)] to be associated with an increase incidence OHCA.^87^ In the second,^49^ TCAs did not increase the risk of OHCA and there were no important differences in risk between individual TCAs (amitriptyline, clomipramine, doxepin, imipramine and nortriptyline). Associations with cardiac arrest are given in Supplemental Table S3.

Supplemental Table S4 gives associations with incident MI. Of the TCAs studied, the following were associated with higher risk for MI: amitriptyline [1.39 (CI: 1.33–1.46)],^85^ dothiepin/dosulepin [1.33 (CI: 1.27–1.38)]^85^ and lofepramine [3.07 (CI: 1.50–6.26), 2.02 (CI: 1.14–3.59),^80^ 1.49 (CI: 1.41–1.59)].^85^

The risk of death associated with clinical doses of individual TCAs was assessed in terms of all-cause mortality (Supplemental Table S5). Association was reported with amitriptyline [1.14 (CI: 1.06–1.21),^78^ 1.10 (CI: 1.03–1.18)],^79^ lofepramine [1.51 (CI: 1.35–1.69)]^79^ and nortriptyline [1.16 (CI: 1.04–1.28)].^78^. Increase in the risk of cardiovascular mortality (Supplemental Table S6) was not linked to any of the TCAs assessed.^78,82^

We identified two studies that assessed the risk of sudden cardiac death or ventricular arrhythmia relative to paroxetine (Supplemental Table S7). In one study, clomipramine was found to have a higher risk than paroxetine [HR 2.71 (CI: 0.62–11.84), 1.26 (CI: 0.28–5.61)].^88^

## Discussion

TCAs differ somewhat in their overdose toxicity, but data presented and summarized here do not support the widely held impression that particular TCAs (e.g. amitriptyline and dothiepin/dosulepin) are uniquely toxic. At one time, dothiepin and amitriptyline together accounted for over 75% of UK antidepressant-related poisoning deaths.^17^ Dothiepin has, in particular, been singled out as having the greatest risk to life in overdose.^89^ Official warnings against their use^90^ ultimately led to a huge decline in prescription numbers^91^ and deaths by dothiepin poisoning.^92^ Data presented here do not support the designation of any particular TCAs (even dothiepin or amitriptyline) as being importantly more toxic in overdose than others, so these warnings may have been misplaced, or at least too limited in scope. All TCAs (with the sole exception of lofepramine) are potentially fatal in overdose and this fact arguably overrides marginal differences between individual drugs. This conclusion has important consequences for the continued widespread use of TCAs for various conditions.

### Fatal toxicity index

The FTI is a measure of recorded deaths set against a denominator that accounts for the extent of use of a particular drug (e.g. number of prescriptions for that drug). In studies assessing mortality following single-drug exposure, desipramine consistently had the highest FTI except where it was prescribed so infrequently that no deaths were reported. Among the other TCAs, amitriptyline and dothiepin/dosulepin had the next highest FTI values, broadly speaking, followed closely by trimipramine, imipramine and doxepin. Nortriptyline, clomipramine and lofepramine seemed to have a lower toxicity in overdose, although there are contradictory data both for clomipramine and, particularly, for nortriptyline. Importantly, however, FTI values were of a similar order of magnitude for all drugs except lofepramine.

In studies including both single- and multiple-dose exposures, FTI values tend to be somewhat higher than those in single-exposure studies. However, the rank order of antidepressants was the same. Again, desipramine had the highest FTI values, whereas lofepramine and clomipramine had very low FTI estimates. Dothiepin/dosulepin and amitriptyline were next to desipramine with relatively high FTI values, followed closely by imipramine and trimipramine. As before, values were of a broadly similar order of magnitude – all TCAs except lofepramine were found to be somewhat toxic in overdose. Differences in overdose toxicity may therefore have little relevance to practice.

FTI values and the rank order for nortriptyline varied remarkably. Nortriptyline was reported to have FTI values similar to amitriptyline when the two substances were found present in blood at the same time (nortriptyline is the major metabolite of amitriptyline). However, in the two studies that excluded mentions where nortriptyline and amitriptyline were both present, nortriptyline FTI values were substantially lower and were similar to lofepramine and clomipramine.^53,60^ Trimipramine also showed some variation. When FTI was defined as deaths per consumption, three studies including trimipramine reported high values compared with the other TCAs included.^65,67,69^ Its FTI was much lower in the studies defining FTI as deaths per million prescriptions (see Tables 1 and 2). It is not clear which of the two methods is the more reliable.

Our assessment of relative risk was of necessity somewhat crude. Because of considerable geographical and temporal overlap and differences in the size (and therefore relative weight) of studies, we were unable to generate summary data such as weighted means or confidence intervals. We were unable to compare any summary FTI values statistically given this overlap in data collection and given the different ways in which studies were conducted (e.g. incorporating deliberate and/or accidental deaths). Our assessment was thus based on a crude examination of the values and consistency or otherwise of values obtained. Having said this, it is clear that, with the exception of lofepramine, all TCAs represent a risk to life when taken in overdose.

The FTI measure was created more than 40 years ago and was initially introduced by Barraclough^93^ as a means to compare mortality rates between hypnotics and barbiturates.^93^ A similar index was proposed the same year by Girdwood but excluded deaths from overdose.^94^ It was then suggested that the FTI measure be extended to cover a wide range of drugs.^59,95^ Differences in the FTI, especially among drugs of the same class, may be a reliable indicator of differences in the inherent toxicity of these drugs.^50,52,56^

As already mentioned, the interpretation of FTI is also complicated by the fact that major metabolites of amitriptyline and imipramine are marketed drugs (nortriptyline and desipramine, respectively). In addition to this, desipramine is the major metabolite of lofepramine. Desipramine has much higher FTI values than all other TCAs. Theoretically, this could be the result of triple counting, as desipramine is also a metabolite of imipramine and lofepramine^96^ and so may be detected at postmortem even when not prescribed. Thus, overdoses of imipramine and lofepramine may be attributed to desipramine in addition to, or even instead of, the parent molecule. However, other sources of data suggest that this is not the case. For example, no death was observed in a review of 55 cases of lofepramine overdoses up to 7.2 g^97^ and lofepramine, when detected alone, has very low FTI values.

### CFR

In accordance with our findings with the FTI, CFR values for desipramine were generally much higher than all other TCAs. Only one study included data on lofepramine and that found no deaths from lofepramine overdose.^72^ Clomipramine also had low CFR values, followed, in increasing rank order, by imipramine and trimipramine and thereafter by doxepin and amitriptyline. Only one study included data on dothiepin/dosulepin and found it to be similarly toxic in overdose to doxepin.^48^ Finally, nortriptyline was again reported to have CFR values similar to amitriptyline when the studies included multiple drug exposures.^26,73^ These data suggest rather lower toxicity for nortriptyline than its parent drug, amitriptyline. Overall, the broad concordance between FTI and CFR findings can be considered an indicator of the reliability of the data summarized here. Again, the broadly similar CFR for all TCAs except lofepramine is the most important observation.

### Stroke in clinical use

In two studies performed by the same research group, no TCA studied (amitriptyline, dothiepin, lofepramine) was shown to increase the risk of stroke relative to periods on no antidepressant in one study^80^ but lofepramine showed an increased risk of stroke in the second.^98^ Imipramine also demonstrated a significantly increased risk of stroke compared to non-use.^86^ Another study that included amitriptyline, melitracen (also a TCA), imipramine and doxepin found a 41% increase in recurrent stroke for patients with any use of TCA.^99^ Oddly, TCA discontinuation was associated with increased risk of stroke^100^ and recurrent stroke,^86^ especially during the first month after discontinuation.

In general, it seems that some TCAs, such as imipramine and doxepin, carry an increased risk for stroke/TIA in clinical use, whereas others, such as amitriptyline, do not.^80,86,98^ Lofepramine also showed a tendency to increase the risk of stroke compared with no antidepressant drug^98^ and was associated with relatively higher relative risk than other TCAs assessed; however, this difference did not reach statistical significance.^80,98^ It should be noted, nonetheless, that statistical association may not be causative (there may be confounding factors) and that the absence of statistical association does not preclude association (there may have been insufficient statistical power to reveal differences).

### Arrhythmia in clinical use

Although certain TCAs such as amitriptyline and imipramine have been associated with arrhythmia risk both in clinical and by mechanistic studies,^101–104^ none of the large observational studies included in this review found any statistically significant association between any of the individual TCAs assessed and the occurrence of arrhythmia (besides an increased risk for lofepramine). This observation perhaps confirms that QT interval prolongation is a poor marker for arrhythmia risk in the case of antidepressants.^80,83^ These findings are also consistent with the suggestion that the increased QT interval recorded for TCAs might be the result of overestimation that occurs in persons with high resting heart rates. When Fridericia’s formula or a model based on regression coefficients was used for QT interval correction in one study, no increase in QTc was observed.^105^

It has been also indicated that even statistically insignificant increases in heart rate could, in the long term, result in significant adverse effects, especially in patients with underlying CVD.^11^ This hypothesis has not been tested in the clinical setting, as the trials identified that assessed the endpoint arrhythmia were not appropriately designed; the only study that investigated arrhythmia in a population of elderly with a history of CVD assessed the association with current use and excluded patients who had used antidepressants within 2 years before cohort entry.^19^ Coupland *et al.*^80^ found no difference in the heart rate (HR) for arrhythmia after 1 *versus* 5 years of follow-up but assessed the risk in a population excluding the elderly. The proportion of patients with comorbid CVD was very low, but, importantly, those prescribed lofepramine did not have a higher likelihood of baseline CVD.^80^ The authors of this study all but ruled out confounding by indication as an explanation for the striking finding with respect to lofepramine, but did note that various risks were higher in patients prescribed lower doses, perhaps indicating that this was not a drug-related effect.

### Out of hospital cardiac arrest

Two studies assessed the risk of OHCA with individual TCAs. In one, the only TCA associated with OHCA was nortriptyline. It was suggested that the widespread off-label use of low-dose amitriptyline could be responsible for the disguising of any potential amitriptyline effect, whereas the lack of an identified effect for imipramine could have been due to the low number of patients receiving treatment with imipramine at the time of OHCA.^87^ Notwithstanding these observations, it is strange that amitriptyline showed no association with this outcome, whereas its major metabolite was linked to an increased risk. Again, statistical association may not be causative, and the absence of statistical association does not preclude association. The second study^49^ found no increased risk for TCAs or any individual TCA (including nortriptyline) and a recent meta-analysis suggests that TCAs as a group may have no adverse effect on the risk of arrhythmia.^106^

### MI in clinical use

Amitriptyline showed an increased risk of MI in one of six studies,^85^ dothiepin in one of four^85^ and lofepramine in three of four.^80,85^ Clomipramine significantly reduced risk in one of four studies.^77^ Overall, these observations suggest no risk for clomipramine, a possible increased risk for amitriptyline and dothiepin and a more probable increased risk for lofepramine. Confounding by indication is again difficult to rule out.

### Mortality in heart failure

In the large-scale study in patients with heart failure,^78^ nortriptyline and amitriptyline were each found to significantly increase all-cause mortality but the increase in cardiovascular mortality did not reach statistical significance. Dothiepin and imipramine were not found to increase the risk of either all-cause or cardiovascular mortality.^78,79,82^ The implication that nortriptyline and amitriptyline are more dangerous than dothiepin and imipramine in clinical use in heart failure is difficult to explain and, as often observed in such studies, may be heavily confounded by other factors.

### Sudden death in clinical use

In the large cohort study of Coupland *et al.*^98^ current use of lofepramine was found to significantly increase the risk of death compared to periods on no antidepressant. Although no direct comparison between individual TCAs was carried out, based on the CIs of the individual TCAs, this increase seems to be greater with lofepramine than with other TCAs assessed (amitriptyline and dosulepin). Amitriptyline also showed a tendency for increased risk.^98^

Amitriptyline, doxepin and nortriptyline were all associated with risks of sudden death/ventricular arrhythmia that were no greater than that of paroxetine, which is considered to have a very favourable cardiovascular profile.^83^ Clomipramine had a much higher HR compared with the other TCAs in this study, but no firm conclusions can be drawn, owing to the low number of events recorded that resulted in wide Cis.^88^

### Mechanism of toxicity

The toxic effects of TCAs are believed to be caused by four main pharmacological properties^107^: inhibition of norepinephrine reuptake; direct α adrenergic block; a membrane stabilizing or quinidine-like effect on the myocardium and anticholinergic action. Accordingly, the main complications of TCA overdose are on the parasympathetic and central nervous system and the cardiovascular system.^102,108–112^

Few studies have sought to identify pharmacological/toxicological reasons behind the differences in mortality rates in overdose. One study found a significant correlation between the FTI of the various TCAs and their ability to decrease cardiac conduction.^50^ Differences in toxicity in overdose cannot be explained based on noradrenaline/serotonin reuptake inhibition selectivity,^50,57,58^ lipid solubility, and potency at histamine H1, muscarinic and α1-adrenergic receptors.^50^ Pharmacogenetic aspects of the pharmacokinetics of each drug are also unlikely to account for the differences seen.^73^

Another study suggested that toxicity may be associated with the interference of each drug with specific membrane-based ion channels and receptors involved in cardiac conduction.^113^ In addition, the degree to which these drugs exert proconvulsant effects has been also suggested to correlate with their inherent toxicity in overdose both directly and indirectly, by increasing cardiac toxicity.^50,53,57,89^ Desipramine, which shows unambiguously higher FTI and CFR values compared with the other TCAs, has been also shown to cause seizures in overdose more frequently than other TCAs.^76,114^

Experimental studies have confirmed that desipramine and amitriptyline have higher membrane-stabilizing activity than both imipramine^115^ and lofepramine.^116^ On the other hand, studies assessing the membrane-stabilizing effects of clomipramine compared with other TCAs have had mixed findings.^113,115,117,118^ The relatively low FTI values for clomipramine in some studies could also be the result of it achieving a greater reduction in suicidality than other drugs: clomipramine is the only TCA that is selective for inhibition of serotonin reuptake, a property which has been associated with a reduction in suicidal ideation.^54^ The increased levels of serotonin resulting from the inhibition of serotonin reuptake have been also associated with a reduction in sympathetic activity that has been shown to protect against arrhythmias.^104^ As discussed above, clomipramine’s main use is obsessive-compulsive disorder (OCD) rather than depression, of course, and this may be another confounding factor.

The increased toxicity of desipramine could be attributed directly to higher toxicity in overdose rather than any confounding factor^112^ although animal studies do not support this hypothesis as desipramine has a much higher median lethal dose (LD_50_) than other TCAs.^54^ However, this increased toxicity in overdose could be an effect uniquely encountered in humans. Another hypothesis is that desipramine might be prescribed to more vulnerable patients due to its comparatively limited adverse effects at therapeutic levels. Finally, it has been also suggested that desipramine could be more readily absorbed in overdose owing to its low degree of anticholinergic activity (an activity that slows gastric emptying).^112^

The lower FTI values of nortriptyline compared with amitriptyline have been suggested to be related to the lower n-octanol/water partition coefficient of nortriptyline (the metabolite is more water soluble than the parent compound). Indeed, the mean blood concentration of nortriptyline in fatal cases is much higher than the corresponding value of amitriptyline perhaps indicating a lower volume of distribution.^59^ The very low FTI values for lofepramine have been proposed to be due to it being more lipophilic than the other TCAs. This feature of lofepramine might result in delayed dissolution and a reduced absorption rate following an overdose.^119^ Lofepramine effectively acts as a prodrug of desipramine. It can also be postulated that a massive overdose might overwhelm the hepatic capacity to convert lofepramine to desipramine. Lofepramine is also preferentially metabolized to didesmethylimipramine^120^ and this may be cardioprotective. Another explanation for this lower FTI for lofepramine could also be that it is prescribed mainly to patients at lower risk of attempting suicide,^69^ although this is a highly unlikely explanation given the dearth of fatalities reported after lofepramine overdose.

The mechanism *via* which TCAs cause their cardiovascular effects is somewhat speculative and surrogate markers such as QT interval prolongation or inhibition of cardiac ion channel currents are not considered appropriate to predict adverse outcomes^80,83^ for clinical use. This is because antidepressants also exert protective effects on the heart rate through the modulation of the cardiac autonomic-mediated physiological responses.^121^ Clinical endpoints were therefore preferred. The adverse cardiovascular events assessed in the present study were heart failure, MI, stroke, TIA, arrhythmia or death. AR, HR and OR are all considered acceptable and widely used measures of treatment effect, especially in relation to risk assessment of adverse events in observational studies.^122–124^

### Limitations

This study has several limitations. All reported measures of toxicity in overdose are contingent on outside influences and confounding. The FTI’s main limitation is that it is only valid for legal, prescription-only medicines and that deaths attributed to a drug used at clinical doses may also be included. Also, sometimes coronial data on the cause of death may be inaccurate, or at least misleading. In addition, the number of prescriptions as a measure of exposure may bring some bias as the choice by the prescriber of a drug for a suicidal patient may take into consideration the safety of drugs in overdose.^48,50,52,54,60,61,67^ Thus, drugs known to be particularly toxic in overdose might be rarely prescribed to suicidal patients, and those that are thought to be non-toxic and effective may be prescribed to more suicidal patients.^125^ This leads to important selection bias and confounding by indication. Licensed indications and preferential use in particular populations may also confer bias; for example, clomipramine is largely used for OCD rather than depression. Other influences on apparent toxicity in overdose include available tablet size (higher dose tablets may be easier to take in overdose quantities), and pack size/prescription duration (more tablets might be available for overdose). We were not able to control these factors, but it is of interest that dothiepin is available as 75 mg tablets^126^ and desipramine is available as 150 mg tablets in the United States^127^ (as is imipramine^128^). The maximum tablet sizes of some other drugs were limited to 25 mg or perhaps 50 mg in some countries.

Further limitations include the previously mentioned unsuitability of FTI and CFR data for presentation as summary means and for the statistical comparison of means, the exclusion of rarely used TCAs from our search and our analysis (although there are in fact very few overdose data on these drugs) and the exclusion of non-English language papers. We had originally intended to include papers not in English but the large variety of languages used in these non-English papers meant translation, even using specialist software, would have been too onerous at the abstract screening phase. We ultimately included 30 papers from 6147 English language papers originally identified (around 0.5%). Using this proportion, we estimate we might have missed perhaps 4 papers in the 787 non-English language papers originally screened. Lastly, an additional limitation is the restricted number of databases screened (we did not include Cochrane or PsycINFO, e.g.). However, we did employ hand searches of all reference sections of all papers included in our analysis and found no additional relevant papers. This might suggest that any remaining studies not found by our search either do not exist or are too obscure to be referenced by authors working in the same narrow field.

CFR is a measure of relative toxicity that aims to determine the risk of death with an agent at overdose (which may be deliberate or accidental). Its major limitation is that it records only cases that reach the hospital or are otherwise attended to and treated. This approach misses both cases of overdose with mild symptoms that may not reach the hospital and fatal cases of home poisoning where hospital treatment is not involved. Thus, drugs that are promptly fatal when taken in overdose may show low CFR as only milder overdoses reach the hospital. Nonetheless, it is interesting to note that a study of outcomes of known suicide attempts, including those not resulting in hospitalization, showed high CFRs for all TCAs included.^75^

## Conclusion

Our analysis confirms that there are only minor differences in the risk of death after an overdose among different TCAs. It may be that desipramine is more likely than other TCAs to lead to death in overdose but the risk is of the same order of magnitude for all but one TCA. Desipramine, amitriptyline, clomipramine, dothiepin, doxepin, imipramine and trimipramine all carry a higher risk for fatalities in overdose than lofepramine. The exact overdose toxicity of nortriptyline is unclear, but its overdose toxicity is of the same order of magnitude for all TCAs except lofepramine.

Evidence to support the possibility that there may be differential cardiotoxicity between individual TCAs in terms of clinical events at clinically relevant exposure remains somewhat elusive. (There is also the possibility that antidepressants as a group confer an increased risk of cardiovascular death in clinical use.^129^) Lofepramine is associated with a significantly increased risk of some adverse cardiovascular events in studies using an overlapping database. This tendency, however, could have been the result of preferential prescribing, rather than reflecting a real difference in cardiotoxicity at clinical doses. It is odd that the least toxic drug in overdose should be identified as having specific clinical use toxicity. Preferential prescribing of lofepramine to high-risk patients seems a credible explanation for these findings. In any case, it seems that the individual risk of causing adverse cardiovascular events in clinical use should not be considered an important factor in deciding which TCA to prescribe. Nor should the clinical toxicity of lofepramine be assumed to be greater than other TCAs.

Given the nature of depression and the high risk of suicide, the risk of death from overdose needs to be weighed against the benefits of each drug to decide on the optimal treatment to prescribe. In this respect, only lofepramine offers an assurance of safety in overdose: all other TCAs are, metaphorically, loaded gun for suicidal individuals. The use of TCAs in conditions other than depression should be carefully considered given TCAs’ shared ability to cause death in overdose and the possibility of increased mortality in clinical use.

## Supplemental Material

sj-docx-1-tpp-10.1177_20451253241243297 – Supplemental material for The cardiovascular safety of tricyclic antidepressants in overdose and in clinical use Supplemental material, sj-docx-1-tpp-10.1177_20451253241243297 for The cardiovascular safety of tricyclic antidepressants in overdose and in clinical use by David Taylor, Sofia Poulou and Ivana Clark in Therapeutic Advances in Psychopharmacology

sj-docx-2-tpp-10.1177_20451253241243297 – Supplemental material for The cardiovascular safety of tricyclic antidepressants in overdose and in clinical use Supplemental material, sj-docx-2-tpp-10.1177_20451253241243297 for The cardiovascular safety of tricyclic antidepressants in overdose and in clinical use by David Taylor, Sofia Poulou and Ivana Clark in Therapeutic Advances in Psychopharmacology

## Declarations

**Ethics approval and consent to participate:** Not applicable.

**Consent for publication:** Not applicable.

**Availability of data and materials:** Not applicable.
