# Supplementary material for: The cardiovascular safety of tricyclic antidepressants in overdose and in clinical use
Source: Ther Adv Psychopharmacol. 2024 May 30;14:20451253241243297. doi: 10.1177/20451253241243297 (PMC11141239; doi:10.1177/20451253241243297)
Supplement: sj-docx-2-tpp-10.1177_20451253241243297 – Supplemental material for The cardiovascular safety of tricyclic antidepressants in overdose and in clinical use [file sj-docx-2-tpp-10.1177_20451253241243297.docx]

**Table S1. Adjusted ORs/HRs for stroke/TIA by TCA^(1)^**

|  |  | **Amitriptyline** | **Dothiepin/**  **dosulepin** | **Doxepin** | **Imipramine** | **Lofepramine** |
| --- | --- | --- | --- | --- | --- | --- |
| Coupland et al 2011[1] ^(3)^ | HR  (95% CI) | 1.01 (0.90-1.13) | 0.95 (0.83-1.09) |  |  | **1.26 (1.02-1.54)** |
| Coupland et al 2016[2] ^(3)^  (1 year) | HR  (95% CI) | 1 (0.59-1.70) | 1.12 (0.63 - 1.98) |  |  | 1.15 (0.43-3.11) |
| Coupland et al 2016[2]^(3)^  (5 years) | HR  (95% CI) | 1.35 (1.00-1.82) | 1.16 (0.77 – 1.76) |  |  | 1.75 (0.96-3.19) |
| Wang et al 2015[3] ^(2)^ (1 year) | OR  (95% CI) | 1 (0.59-1.71) |  | 1.68 (0.85-3.32) | **1.41 (1.13-1.76)** |  |

^(1)^ Clomipramine, desipramine, nortriptyline and trimipramine were omitted as no data were available for these drugs

^(2)^ Single-drug exposure

^(3)^ Both single- and multiple-drug exposure

Abb: OR: odd ratio; HR – hazard ratio; TIA – transient ischaemic attack; CI – confidence interval

| **Study** | Time period | Country |
| --- | --- | --- |
| Coupland et al 2011 | 1996-2008 | UK |
| Coupland et al 2016 | 2000-2011 | UK |
| Wang et al 2015 | 2000-2008 | Taiwan |

Shading = overlap in data collection period

**Table S2. Adjusted ORs/HRs for arrhythmia by TCA^(1)^**

|  |  | **Amitriptyline** | **Dothiepin/dosulepin** | **Lofepramine** |
| --- | --- | --- | --- | --- |
| Biffi et al 2018[4] ^(2)^ (15 days) | OR (95% CI)  nested-case control design | 1.49 (0.71-3.14) |  |  |
|  | OR (95% CI)  case-crossover design | 0.80 (0.24-2.73) |  |  |
| Coupland et al 2016^[2] (2)^  (1 year) | HR (95% CI) | 1.15 (0.69-1.94) | 0.73 (0.35-1.50) | **2.13 (1.05-4.33)** |
| Coupland et al 2016[2] ^(2)^  (5 years) | HR (95% CI) | 1.16 (0.87-1.54) | 0.93 (0.61-1.40) | **1.67 (1.01-2.76)** |

^(1)^ Clomipramine, desipramine, doxepin, imipramine, nortriptyline and trimipramine were omitted as no data were available for these drugs

^(2)^ Both single- and multiple-drug exposure

Abb: OR: odd ratio; HR – hazard ratio; CI – confidence interval

| **Study** | Time period | Country |
| --- | --- | --- |
| Biffi et al | 2008-2010 | Italy |
| Coupland et al 2011 | 1996-2008 | UK |
| Coupland et al 2016 | 2000-2011 | UK |

Shading = overlap in data collection period

**Table S3. Adjusted ORs for cardiac arrest by TCA^(1)^**

| **Reference** | **Measure** | **Amitriptyline** | **Clomipramine** | **Doxepine** | **Imipramine** | **Nortriptyline** |
| --- | --- | --- | --- | --- | --- | --- |
| Eroglu et al 2022[5] | HR (95% CI) | Ref. | 0.70 (0.46-0.97) | 0.81 (0.52-1.27) | 1.00 (0.75-1.33) | 0.98 (0.77-1.24) |
| Weeke et al 2012[6]^(2)^ | OR (95% CI) | 1.36 (0.83−2.32) | - | - | 0.95 (0.35−2.56) | **5.14 (2.17−12.2)** |

^(1)^ Desipramine, dothiepin/dosulepin, lofepramine and trimipramine were omitted as no data were available for these drugs

^(2)^ Both single- and multiple-drug exposure

Abb; HR- hazard ratio, OR – odds ratio

**Table S4. Adjusted ORs/HRs for MI by TCA^(1)^**

| **Reference** | **Outcome** | **Amitriptyline** | **Clomipramine** | **Dothiepin/**  **dosulepin** | **Doxepin** | **Imipramine** | **Lofepramine** |
| --- | --- | --- | --- | --- | --- | --- | --- |
| **Alqdwah-Fattouh et al 2020[7]** | HR (95% CI) | 0.95  (0.80-1.14) | **0.62**  **(0.40-0.96)** |  |  | 1.57  (0.79-3.11) |  |
| **Coupland et al 2011[1] ^(2)^** | HR (95% CI) | 1.1  (0.93-1.31) |  | 1.07  (0.88-1.29) |  |  | 1.18  (0.86-1.61) |
| **Coupland et al 2016[2] ^(2)^ (1 year)** | HR (95% CI) | 0.75  (0.37-1.55) |  | 1.07  (0.53-2.18) |  |  | **3.07**  **(1.50-6.26)** |
| **Coupland et al 2016[2] ^(2)^ (5 years)** | HR (95% CI) | 1.17  (0.82-1.66) |  | 1.17  (0.75-1.83) |  |  | **2.02**  **(1.14-3.59)** |
| **Tata et al 2005[8] ^(2)^** | OR (95% CI) | **1.39**  **(1.33-1.46)** |  | **1.33**  **(1.27-1.38)** |  |  | **1.49**  **(1.41-1.59)** |
| **Wu et al 2017a ^(2)^** | OR (95% CI) | 0.76  (0.40-1.44) | 0.48  (0.05-4.41) |  | 0.93  (0.42-2.05) | 1.11  (0.85-1.45) |  |

^(1)^ Desipramine, nortriptyline and trimipramine were omitted as no data were available for these drugs

^(2)^ Both single- and multiple-drug exposure

Abb: OD – odds ratio; HR- hazard ratio; MI – myocardial infarction; CI – confidence interval

| **Study** | Time period | Country |
| --- | --- | --- |
| Alqdwah-Fattouh et al 2020 | 2002-2015 | Spain |
| Coupland et al 2011 | 1996-2008 | UK |
| Coupland et al 2016 | 2000-2011 | UK |
| Tata et al 2005 | 1988-2001 | UK |
| Wu et al 2017 | 2001-2012 | Taiwan |

Shading = overlap in data collection period

**Table S5. Adjusted ORs/HRs for all-cause mortality by TCA^(1)^**

|  |  | **Amitriptyline** | **Clomipramine** | **Dothiepin/dosulepin** | **Doxepin** | **Imipramine** | **Lofepramine** | **Nortriptyline** |
| --- | --- | --- | --- | --- | --- | --- | --- | --- |
| **Brouwers et al 2016[9] ^(2)^** | HR (95% CI) | **1.14**  **(1.06–1.21)**  **P <0.001** |  | 0.95 (0.68–1.32)  P 0.74 |  | 1.04  (0.90–1.21)  P 0.06 |  | **1.16**  **(1.04–1.28)**  **P 0.005** |
| **Coupland et al 2011[1] ^(2)^** | HR (95% CI) | **1.10**  **(1.03-1.18)** |  | 1.03 (0.95-1.13) |  |  | **1.51**  **(1.35-1.69)** |  |
| **Danielsson et al 2016[10] ^(2)^** | OR (95% CI) | 1.10 |  |  |  |  |  |  |

^(1)^ Desipramine and trimipramine were omitted as no data were available for these drugs

^(2)^ Both single- and multiple-drug exposure

Abb: OD – odds ratio; HR- hazard ratio; CI – confidence interval

| **Study** | Time period | Country |
| --- | --- | --- |
| Brouwers et al 2016 | 1997-2010 | Denmark |
| Coupland et al 2011 | 1996-2008 | UK |
| Danielsson et al 2016 | 2008-2013 | Sweden |

**Table S6. Adjusted ORs/HRs for cardiovascular mortality by TCA^(1)^**

|  |  | **Amitriptyline** | **Clomipramine** | **Dothiepin/dosulepin** | **Doxepin** | **Imipramine** | **Nortriptyline** |
| --- | --- | --- | --- | --- | --- | --- | --- |
| Brouwers et al 2016^[9]^ ^(2)^ | HR | 1.08 (0.99–1.17)  P 0.09 |  | 0.60 (0.38–0.93)  P 0.02 |  | 0.97 (0.81–1.16)  P 0.72 | 1.11 (0.97–1.26)  P 0.12 |
| Jolly et al 2009[11] ^(2)^ | OR | 1.17 (0.64-2.12) | 1.63 (0.28-9.35) |  |  | 0.63 (0.07-5.36) |  |

^(1)^ Desipramine, lofepramine and trimipramine were omitted as no data were available for these drugs

^(2)^ Both single- and multiple-drug exposure

Abb: OD – odds ratio; HR- hazard ratio; CI – confidence interval

| **Study** | Time period | Country |
| --- | --- | --- |
| Brouwers et al 2016 | 1997-2010 | Denmark |
| Jolly et al 2009 | 2003-2007 | UK |

**Table S7. Comparative safety of antidepressants relative to paroxetine with regard to serious arrhythmia recorded as sudden cardiac death or ventricular arrhythmia ^(1)^**

|  |  | **Amitriptyline** | **Clomipramine** | **Desipramine** | **Dothiepin/**  **dosulepin** | **Doxepin** | **Imipramine** | **Nortriptyline** |
| --- | --- | --- | --- | --- | --- | --- | --- | --- |
| **Leonard et al 2011[12]** ^(2)^ | Adjusted HR | 1.0 (NR) |  |  |  | 1.2 (NR) |  | 1.1 (NR) |
| **Wu et al 2017b[13]** ^(2)^ | Adjusted Crude Cox Regression Model (95% CI) | 0.69  (0.16-3.01) | 2.71  (0.62-11.84) |  | NA | 0.78  (0.18-3.41) | 0.96  (0.43-2.14) |  |
|  | Adjusted Propensity Score Stratification (95% CI) | 0.45  (0.10-2.03) | 1.26  (0.28-5.61) |  | NA | 0.41  (0.09-1.83) | 0.53  (0.23-1.2) |  |

^(1)^ Lofepramine was omitted as no data were available for it ^(2)^ Both single- and multiple-drug exposure

Abb: OD – odds ratio; HR- hazard ratio; MI – myocardial infarction; CI – confidence interval; NR -not recorded

| **Study** | Time period | Country |
| --- | --- | --- |
| Leonard et al 2011 | 1999-2003 | US |
| Wu et al 2017 | 2001-2012 | Taiwan |

**References**

1. Coupland C, Dhiman P, Morriss R, Arthur A, Barton G, Hippisley-Cox J. Antidepressant use and risk of adverse outcomes in older people: population based cohort study. BMJ. 2011 2011;343:d4551.

2. Coupland C, Hill T, Morriss R, Moore M, Arthur A, Hippisley-Cox J. Antidepressant use and risk of cardiovascular outcomes in people aged 20 to 64: cohort study using primary care database. BMJ. 2016 Mar 22;352:i1350.

3. Wang MT, Chu CL, Yeh CB, Chang LC, Malone DC, Liou JT. Antidepressant use and risk of recurrent stroke: a population-based nested case-control study. J Clin Psychiatry. 2015 Jul;76(7):e877-85.

4. Biffi A, Rea F, Scotti L, Mugelli A, Lucenteforte E, Bettiol A, et al. Antidepressants and the risk of arrhythmia in elderly affected by a previous cardiovascular disease: a real-life investigation from Italy. Eur J Clin Pharmacol. 2018 Jan;74(1):119-29.

5. Eroglu TE, Barcella CA, Gerds TA, Kessing LV, Zylyftari N, Mohr GH, et al. Risk of out-of-hospital cardiac arrest in antidepressant drug users. Br J Clin Pharmacol. 2022 Jul;88(7):3162-71.

6. Weeke P, Jensen A, Folke F, Gislason GH, Olesen JB, Andersson C, et al. Antidepressant use and risk of out-of-hospital cardiac arrest: a nationwide case-time-control study. Clin Pharmacol Ther. 2012 Jul;92(1):72-9.

7. Alqdwah-Fattouh R, Rodríguez-Martín S, de Abajo FJ, González-Bermejo D, Gil M, García-Lledó A, et al. Differential effects of antidepressant subgroups on risk of acute myocardial infarction: A nested case-control study. Br J Clin Pharmacol. 2020 Oct;86(10):2040-50.

8. Tata LJ, West J, Smith C, Farrington P, Card T, Smeeth L, et al. General population based study of the impact of tricyclic and selective serotonin reuptake inhibitor antidepressants on the risk of acute myocardial infarction. Heart. 2005 4/2005;91(4):465-71.

9. Brouwers C, Christensen SB, Damen NL, Denollet J, Torp-Pedersen C, Gislason GH, et al. Antidepressant use and risk for mortality in 121,252 heart failure patients with or without a diagnosis of clinical depression. Int J Cardiol. 2016 Jan 15;203:867-73.

10. Danielsson B, Collin J, Jonasdottir Bergman G, Borg N, Salmi P, Fastbom J. Antidepressants and antipsychotics classified with torsades de pointes arrhythmia risk and mortality in older adults - a Swedish nationwide study. Br J Clin Pharmacol. 2016 Apr;81(4):773-83.

11. Jolly K, Gammage MD, Cheng KK, Bradburn P, Banting MV, Langman MJ. Sudden death in patients receiving drugs tending to prolong the QT interval. Br J Clin Pharmacol. 2009 Nov;68(5):743-51.

12. Leonard CE, Bilker WB, Newcomb C, Kimmel SE, Hennessy S. Antidepressants and the risk of sudden cardiac death and ventricular arrhythmia. Pharmacoepidemiol Drug Saf. 2011 Sep;20(9):903-13.

13. Wu CS, Wu HT, Tsai YT, Huang YW, Tsai HJ. Use of antidepressants and risk of hospitalization for acute myocardial infarction: A nationwide case-crossover study. J Psychiatr Res. 2017 Nov;94:7-14.
